# Low-dose-intensity bevacizumab with weekly irinotecan for platinum- and taxanes-resistant epithelial ovarian cancer

**DOI:** 10.1007/s00280-015-2680-4

**Published:** 2015-01-20

**Authors:** Ying Liu, Zhonghai Ren, Shuning Xu, Hua Bai, Ning Ma, Feng Wang

**Affiliations:** 1 Department of Oncology, Henan Cancer Hospital, Zhengzhou University Affiliated Cancer Hospital, Zhengzhou, Henan China; 2 Department of Oncology, Nanyang City Center Hospital, Nanyang, Henan China; 3 Department of Oncology, People’s Hospital of Zhengzhou, Zhengzhou, Henan China; 4 Department of Oncology, Henan Provincial People’s Hospital, Zhengzhou, Henan China; 5 Department of Oncology, The First Affiliated Hospital of Zhengzhou University, Zhengzhou, Henan China

**Keywords:** Bevacizumab, Irinotecan, Platinum-/taxanes-resistant, Ovarian cancer

## Abstract

**Purpose:**

The purpose of this study was to evaluate the safety and efficacy of low-dose-intensity bevacizumab and weekly irinotecan as salvage treatment for patients with platinum- and taxanes-resistant advanced epithelial ovarian cancer.

**Methods:**

Fifty-two patients with platinum- and taxanes-resistant advanced epithelial ovarian cancer received bevacizumab 5 mg/Kg days 1 and 15; irinotecan 60 mg/m^2^ days 1, 8 and 15. The combined therapy was repeated every 28 days, up to six cycles.

**Results:**

A total of 230 cycles of bevacizumab combined with irinotecan were administrated to 52 patients. Among the 52 patients, 22 patients achieved partial response (42.3 %); 12 patients had stable disease (23.1 %) and 18 patients experienced disease progression (34.6 %). The median progression-free survival and the median overall survival were 8.0 months (95 % confidence interval: 6.74–9.26 months) and 13.8 months (95 % confidence interval: 11.97–15.63 months), respectively. The most frequent grade 3–4 hematologic toxicities were neutropenia (11.5 %) and thrombocytopenia (3.8 %). The non-hematologic toxicities included grade 3 diarrhea (3.8 %) and hypertension (3.8 %). Two patients (3.8 %) were confirmed with deep vein thrombosis. Febrile neutropenia, symptomatic cardiac dysfunction and gastrointestinal perforation were not observed in this study.

**Conclusions:**

The combination of low-dose-intensity bevacizumab and weekly irinotecan was an effective and safe regimen for patients with platinum- and taxanes-resistant epithelial ovarian cancer.

## Introduction

Ovarian cancer is the leading cause of death in women with gynecological cancer. Each year, over 2,25,000 cases are diagnosed, and about 1,40,200 women die of the disease worldwide [1]. More than 70 % of patients are diagnosed with advanced ovarian cancer, and the prognoses for these patients are very poor. The 5-year survival rate for stage III cases is 23–41 %, and it is only 11 % for stage IV cases [2]. Currently, surgical tumor debulking followed by platinum- and taxanes-based chemotherapy is the standard therapy for advanced ovarian cancer [3]. The objective response rate (ORR) of the standard therapy for ovarian cancer exceeds 80 %. However, 70 % of patients who have received cytoreduction and chemotherapy will experience recurrence within 5 years due to development of resistance to platinum and taxanes [4]. Patients who suffer disease relapse within 6 months after platinum- or taxanes-containing therapy are considered to be platinum or taxanes resistant. Previous studies have shown that combination chemotherapy increased drug-related toxicity and has no survival benefit to patients with platinum- and taxanes-resistant advanced epithelial ovarian cancer [5, 6]. Nowadays, the standard salvage treatment for these patients is monotherapy without platinum and taxanes. For instance, topotecan [7], liposomal doxorubicin [8], gemcitabine [9] and vinorelbine [10] have all been utilized in practice. The ORR of these single chemotherapy regimens ranges from 10 to 30 % without survival benefit. Therefore, novel treatment strategies are needed to improve the overall survival (OS) of patients with platinum- and taxanes-resistant advanced ovarian cancer.

In addition to traditional cytotoxic chemotherapeutics, molecular targeted drugs have emerged as important weapons in the arsenal of cancer treatment. Anti-angiogenesis prevents the growth of blood vessels that feed tumors and has been proven to be one of the most vital pathways for targeted cancer therapies. In ovarian cancer, over expression of vascular endothelial growth factor (VEGF) has been observed and several studies have verified that high level of VEGF not only increases the risk of ascites but is also closely related to the poor prognosis [11–13]. Therefore, angiogenesis is thought to be a promising target for treating ovarian cancer. Bevacizumab is a recombinant humanized monoclonal IgG1 antibody which targets vascular endothelial growth factor (VEGF)-A. IgG1 antibody is able to inhibit tumor growth and metastasis by binding to and neutralizing all biologically active forms of VEGF-A [14]. Furthermore, anti-VEGF drugs such as bevacizumab are thought to enhance the effect of chemotherapeutic agents by normalization of tumor vessels, leading to increased tumor oxygenation and better delivery of cytotoxic drugs [15].

Bevacizumab (either alone or combined with chemotherapy) has been shown to be effective for patients with recurrent ovarian cancer. Two early phase II studies of bevacizumab monotherapy at a dose of 15 mg/Kg/3 weeks for patients with relapsing ovarian cancer achieved an ORR of 15.9–21 % [16, 17]. In addition, several other phase II studies evaluated the safety and activity of bevacizumab of 10 mg/Kg/2 weeks or 15 mg/Kg/3 weeks in combination with different cytotoxic agents in advanced ovarian cancer [18–20]. These results demonstrated that high-dose-intensity (5 mg/Kg/week) bevacizumab combined with chemotherapy improved the ORR and prolonged the progression-free survival (PFS) of patients with platinum- and taxanes-resistant ovarian cancer. However, these high-dose-intensity bevacizumab (5 mg/Kg/week) combination regimens resulted in more severe adverse effects than the single agents, especially increasing the incidence of bevacizumab-related gastrointestinal perforation and cardiovascular toxicities [21]. Encouragingly, a 2014 phase III study investigating similar doses of bevacizumab combined with chemotherapy for platinum-resistant ovarian cancer has reported a low GI perforation (non-fatal and fatal) of 2.2 % in the AURELIA study [22].

In this study, we aim to develop a safer yet still effective bevacizumab-based combination therapy. Thus, we reasoned that lowering the dose of bevacizumab in conjunction with a carefully chosen chemotherapy might achieve a balance between efficacy and toxicity. In fact, beneficial effects of lower dose-intensity of bevacizumab (2.5 mg/Kg/week) have been shown in a recent single-agent study in relapsing ovarian cancer [23]. In another phase III trial ICON7, the dose of 7.5 mg/Kg/3 weeks as first-line treatment in ovarian cancer was shown to be much safer with risk of 1 % GI perforation [24]. In terms of selection of chemotherapy agent, we decided to choose irinotecan, which has demonstrated positive effect in numerous cancer therapies. The ORR of single irinotecan for platinum- and taxanes-resistant ovarian cancer is 29 % with good tolerance [25]. In addition, different dosing schedules have been developed for irinotecan, and the weekly administration regimen showed moderate efficacy with fewer cases of myelosuppression and diarrhea [26]. The proven clinical activities of bevacizumab and irinotecan as monotherapy, the potentially synergistic anti-angiogenic effects and their non-overlapping toxicity profiles all provide compelling rationale for combining the two drugs for patients with advanced ovarian cancer. Currently, no study of bevacizumab combined with irinotecan for advanced ovarian cancer has been documented. Therefore, we designed this study to evaluate the safety and efficacy of low-dose-intensity bevacizumab and weekly irinotecan as salvage chemotherapy for patients with platinum- and taxanes-resistant advanced ovarian cancer.

## Methods

### Study design

This study was a phase II trial and was designed to evaluate the efficacy and safety of six cycles of bevacizumab plus irinotecan in platinum- and taxanes-resistant advanced ovarian cancer patients. This study was initiated in November 2010 and completed enrollment in May 2013. Local ethics committee approval was obtained before any patient was enrolled into the study which was performed in accordance with the Declaration of Helsinki. All patients signed informed consent before study entry.

### Patients’ eligibility

Eligibility criteria for study entry were as follows: patients were at least 18 years of age with cytologically or histologically proved epithelial ovarian or fallopian tube cancer. All patients received prior primary platinum- and taxanes-based first-line chemotherapy. Patients required measurable disease according to response evaluation criteria in solid tumors (RECIST) [27], Eastern Cooperative Oncology Group (ECOG) performance status ≤2, adequate bone marrow (absolute neutrophil count ≥1.5 × 10^9^/L, platelet count ≥100 × 10^9^/L, hemoglobin ≥90 g/L), normal hepatic function (bilirubin ≤1.5 × the upper limit of normal, alanine aminotransferase and aspartate aminotransferase ≤2.5 × the upper limit of normal), normal renal function(serum creatinine ≤1.5 × the upper limit of normal or creatinine clearance >60 ml/min), normal cardiac function. Furthermore, patients had life expectancy ≥12 weeks. All patients signed informed consent before treatment.

Exclusion criteria included a concurrent malignancy; significant medical comorbidities; clinically significant cardiovascular disease including uncontrolled hypertension, myocardial infarction and unstable angina; New York Heart Association grade 2 or greater congestive heart failure; history of active bleeding; major surgery within 28 days before the initiation of study treatment; serious infections, non-healing wound, ulcer or bone fracture; pregnancy or lactation; known central nervous system metastases; bowel obstruction; and inability to sign informed consent. Patients who have been exposed to topotecan, irinotecan or bevacizumab were excluded.

### Treatment and dose modification

Eligible patients received bevacizumab (Avastin: Roche, Basel, Switzerland) 5 mg/Kg days 1 and 15; irinotecan (Campto: Pfizer, New York, NY, U.S.A.) 60 mg/m^2^ days 1, 8 and 15. The dose cycles were repeated every 28 days for a maximum of six cycles. Bevacizumab was given by intravenous over 90 min for the first time and 30 min for subsequent infusion. Irinotecan was administered intravenously over 60–90 min.

There was no dose modification of bevacizumab. According to the adverse events, bevacizumab was either given or withheld. If the patients experienced severe bevacizumab-related toxicities such as symptomatic cardiac dysfunction, uncontrolled hypertension grade 4, hemorrhage grade 3–4, grade 4 proteinuria, any grade of arterial thrombosis and gastrointestinal perforation, they were removed from the study. Dose of irinotecan was reduced sequentially to 50 mg/m^2^ and to 40 mg/m^2^ for grade 3–4 toxicities or for a treatment-related toxicity causing delay in treatment. Irinotecan was omitted if patients had hematologic toxicity of grade 4 or non-hematologic toxicity grade ≥3 on the day of the schedule administration. If patients experienced toxicity grade ≥3 after two dose reduction of irinotecan, they were withdrawn from the study. Treatment did not stop until one of the following criteria was met: disease progression, unacceptable toxicity, patient refusal, or need to delay chemotherapy more than 3 weeks.

### Response and toxicity assessment

Patients were considered assessable if they had completed at least two cycles of chemotherapy or were removed from study due to disease progression. Baseline evaluation consisted of a complete history and physical examination that included a gynecological examination, laboratory studies including CA-125 marker analysis and dynamic contrast-enhanced computed tomography (CT) or magnetic resonance imaging (MRI) scans within 4 weeks of study entry. Tumor objective response was evaluated every two cycles during therapy and every 8 weeks during follow-up according to RESIST [27] by dynamic contrast-enhanced CT or MRI scans, including the confirmation of response. The level of CA-125 change was not used as the standard for response evaluation. All patients who received at least one dose of study treatment were evaluated for toxicity. Toxicities were assessed and graded according to common terminology criteria for adverse events (NCI-CTCAE) version 4.0 [28] every dose cycle.

### Statistical analysis

The primary endpoints of this phase II study were safety and PFS; the secondary endpoints were OS and ORR. Based on the recent studies of platinum- and taxanes-resistant recurrent epithelial ovarian cancer, the null hypothesis set a PFS of 4.5 months for non-platinum chemotherapy without bevacizumab. The alternative hypothesis proposed a PFS of 7.5 months. A two-sided log-rank test with a sample size of 46 assessable patients achieves 80 % power at 5 % type I error level to reject the null hypothesis. Progression-free survival was measured from the date of enrollment to the date of the first observation of disease progression or the date of death for any cause if there had been no progression. Patients without disease progression at the time of analysis or at time of death were censored at their last date of efficacy evaluation. Overall survival was measured from the date of enrollment until the date of death for any cause. Overall survival was censored at the last date of follow-up. PFS and OS curves were calculated using the Kaplan–Meier method.

## Results

### Patient characteristics

From November 2010 to May 2013, a total of 52 patients were recruited. All the patients were evaluated for toxicity and efficacy. Demographics are listed in Table 1. The median age was 55 years (range 33–65 years), and the median ECOG performance status was 0 (range 0–2). Forty-two of 52 patients (80.8 %) experienced surgical tumor debulking. The median number of prior chemotherapy regimen was 2 (range 1–3).

**Table 1:** Patient demographics

Demographics	Bevacizumab and irinotecan *N* = 52
Median age (years)	55 (33–65)
ECOG performance status	
0	36 (69.2)
1	12 (23.1)
2	4 (7.7)
Primary site, no (%)	
Fallopian tube	8 (15.4)
Ovarian	44 (84.6)
Histology, no (%)	
Serous	38 (73.1)
Endometrioid	8 (15.4)
Mixed pattern	6 (11.5)
Disease stage, no (%)	
II	12 (23.1)
III	26 (50.0)
IV	14 (26.9)
Cytoreductive surgery, no (%)	
Yes	42 (80.8)
No	10 (19.2)
Prior regimen, no (%)	
1	14 (27.0)
2	32 (61.5)
3	6 (11.5)

*ECOG* Eastern Cooperative Oncology Group

### Clinical efficacy

**Fig. 1:**
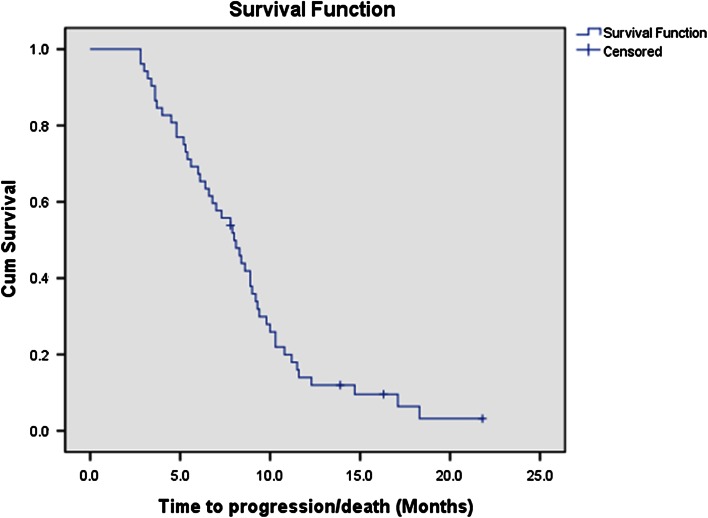
Progression-free survival of patients with platinum- and taxanes-resistant advanced epithelial ovarian cancer received bevacizumab and irinotecan (median progression-free survival: 8.0 months [95 % confidence interval: 6.74–9.26 months])

**Fig. 2:**
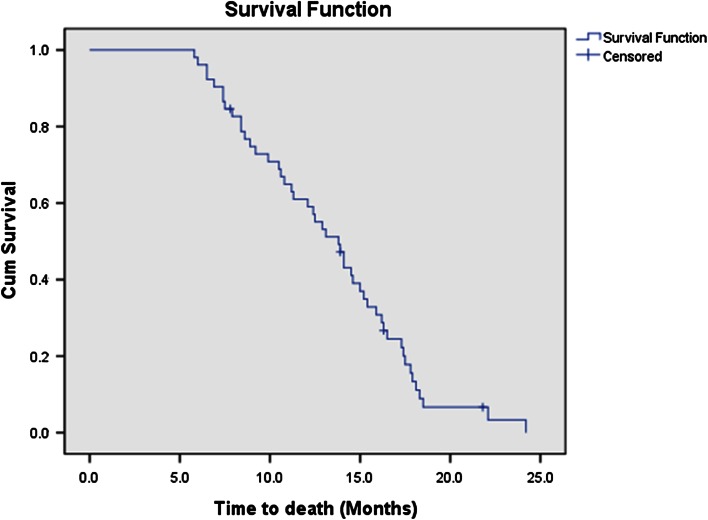
Overall survival of patients with platinum- and taxanes-resistant advanced epithelial ovarian cancer received bevacizumab and irinotecan (median overall survival: 13.8 months [95 % confidence interval: 11.97–15.63 months])

Fifty-two eligible patients received 230 cycles of bevacizumab plus irinotecan treatment in all. None of the patients achieved complete response, 22 patients experienced partial response (42.3 %), 12 patients had stable disease (23.1 %), and the other 18 patients were confirmed with progressive disease (34.6 %). The ORR was 42.3 %, and disease control rate was up to 65.4 % (Table 2). A total of 48 deaths were observed among the 52 patients during or after the therapy. The median PFS (Fig. 1) and median OS (Fig. 2) were 8.0 months (95 % confidence interval: 6.74–9.26 months) and 13.8 months (95 % confidence interval: 11.97–15.63 months), respectively.

**Table 2:** Objective response

Bevacizumab and irinotecan		
Response	Number of patients (*N* = 52)	%
Complete response	0	0
Partial response	22	42.3
Stable disease	12	23.1
Progressive disease	18	34.6
Disease control rate	65.4	
Overall response rate	42.3

### Toxicity

All of the 52 patients who received the bevacizumab plus irinotecan therapy were assessable for safety analysis. There was no therapy-related death. Table 3 lists the most common adverse events. The grade 3 hematologic toxicities were neutropenia (11.5 %) and thrombocytopenia (3.8 %).Grade 4 hematologic toxicity was not observed in our study. Among the 52 patients, two patients (3.8 %) experienced grade 3 diarrhea with cramp. two patients (3.8 %) received grade 3 hypertension, and two patients (3.8 %) was confirmed with deep vein thrombosis after four cycles of combination therapy. There was no febrile neutropenia, symptomatic cardiac dysfunction, grade 4 hypertension, hemorrhage or gastrointestinal perforation.

**Table 3:** Treatment-related adverse events

Adverse events	Bevacizumab and irinotecan (*N* = 52)			
	Grade 1 *N* (%)	Grade 2 *N* (%)	Grade 3 *N* (%)	Grade 4 *N* (%)
Hematologic				
Neutropenia	8 (15.4)	6 (11.5)	6 (11.5)	0
Anemia	4 (7.7)	2 (3.8)	0	0
Thrombocytopenia	4 (7.7)	0	2 (3.8)	0
Febrile neutropenia	0	0	0	0
Non-hematologic				
Gastrointestinal disorders				
Nausea	6 (11.5)	4 (7.7)	0	0
Vomiting	2 (3.8)	2 (3.8)	0	0
Cramp	2 (3.8)	0	2 (3.8)	0
Constipation	10 (19.2)	2 (3.8)	0	0
Diarrhea	4 (7.7)	2 (3.8)	2 (3.8)	0
Ileus	0	0	0	0
Gastrointestinal perforation	0	0	0	0
Neurotoxicity				
Headache	6 (11.5)	0	0	0
Sensory nerve disorder	4 (7.7)	0	0	0
Renal and urinary disorders				
Proteinuria	2 (3.8)	0	0	0
Renal failure acute	0	0	0	0
Cardiac and Vascular disorders				
Asymptomatic decrease in ejection fraction	2 (3.8)	0	0	0
Symptomatic cardiac dysfunction	0	0	0	0
Hypertension	6 (11.5)	2 (3.8)	2 (3.8)	0
Deep vein thrombosis	2 (3.8)			
Arterial thrombosis	0			
Others				
Fatigue	6 (11.5)	0	0	0
Appetite loss	4 (7.7)	2 (3.8)	0	0
Alopecia	8 (15.4)	4 (7.7)	0	0

## Discussion

As suggested by our data presented above, the combination of low-dose-intensity bevacizumab (5 mg/Kg/2 weeks) and weekly irinotecan (60 mg/m^2^ d1, 8, 15 q28d) seems to be an effective treatment with moderate toxicity in patients with platinum- and taxanes-resistant advanced epithelial ovarian cancer.

In terms of efficacy, our combination protocol displayed an ORR of 42.3 % with a disease control rate of 65.4 %, a median PFS of 8.0 months (95 % confidence interval: 6.74–9.26 months) and a median OS of 13.8 months (95 % confidence interval: 11.97–15.63 months). These results have shown significant improvement compared with single bevacizumab or irinotecan regimens. In a recent review of anti-angiogenic agents, single bevacizumab (15 mg/Kg/3 weeks) in recurrent ovarian cancer gave an ORR of 16–21 % with a median PFS of 4.4–4.7 months [29]. As for the single irinotecan regimen in patients with platinum-resistant ovarian cancer, a phase II study demonstrated that irinotecan of 300 mg/m^2^/3 weeks had an ORR of 17.2 % and a median PFS of 2.8 months [30].

As mentioned earlier, bevacizumab has been combined with different cytotoxic agents for treatment of platinum- and taxanes-resistant ovarian cancer. In all of these studies, bevacizumab was administered at higher dosage than that used in our treatment plan (5 mg/Kg/2 weeks). For example, in a single-arm phase II study, 44 patients were treated with bevacizumab at the dose of 15 mg/Kg/3 weeks in combination with liposomal doxorubicin, yielding an ORR of 30.2 % and a median PFS of 7.8 months [20], while in another phase II study, 10 mg/Kg/2 weeks of bevacizumab plus topotecan received 25 % ORR with a median PFS of 7.8 months [18]. In the phase III AURELIA study, the same dose of bevacizumab plus chemotherapy in platinum-resistant ovarian cancer received 27.3 % ORR with a median PFS of 6.7 months [22]. These results showed comparable survival benefits as those observed in our study.

Safety, as well as efficacy, is critical for patients who have received heavy chemotherapy to participate the salvage treatment. In our study, the grade 3 hematologic toxicities were neutropenia (11.5 %) and thrombocytopenia (3.8 %). Grade 3–4 non-hematologic toxicities were diarrhea (3.8 %) and hypertension (3.8 %). In addition, two patients were confirmed with deep vein thrombosis after four cycles of combination therapy. Among the 52 patients, grade 4 hematologic toxicity and bevacizumab-related adverse events such as gastrointestinal perforation, cardiac dysfunction, arterial thrombosis, grade 4 proteinuria and hypertension were not observed.

In the study of single bevacizumab, 44 patients who received 15 mg/Kg/3 weeks of bevacizumab experienced severe adverse events [17]. Bevacizumab-related grade 3–4 toxicities including hypertension (9.1 %), proteinuria (15.9 %) and bleeding (2.3 %) were observed during the therapy. Furthermore, arterial thromboembolic events occurred in three patients (6.8 %), and five patients (11.4 %) experienced gastrointestinal perforation. Eventually, high-dose-intensity bevacizumab resulted in the death of three patients [17]. The regimen of high-dose-intensity of bevacizumab (15 mg/Kg/3 weeks) combined with liposomal doxorubicin resulted in occurrence of more grade 3–4 adverse events including palmar and plantar erythroderma (52 %), hypertension (46 %), headache (11.4 %) and pruritus (9.1 %). As is well known, the toxicity that should raise more concern is cardiac dysfunction. In the study, seven patients (16 %) experienced an asymptomatic decrease in ejection fraction. Finally, a total of 29.5 % of patients did not tolerate the 15 mg/Kg/3 weeks bevacizumab dosage and discontinued the study [20]. These studies demonstrated that high-dose-intensity of bevacizumab in combination with cytotoxic regimens displayed high risk of severe adverse events in patients with platinum-resistant advanced ovarian cancer. In our studies, low-dose-intensity of bevacizumab combined with irinotecan has greatly lowered bevacizumab-related toxicity while in the meantime shown good efficacy compared with previous phase II studies.

## Conclusions

The combination of low-dose-intensity bevacizumab and weekly irinotecan provided significant efficacy with good tolerance in patients with platinum- and taxanes-resistant ovarian cancer. The results of the combination therapy warrant further investigation of a larger randomized phase III trial. Attention should be given to determine the optimal dosing schedule and to minimize treatment-related toxicities.
